# Supplementary material for: Proteomic Analysis Reveals the Effects of Different Dietary Protein Levels on Growth and Development of Jersey-Yak
Source: Animals (Basel). 2024 Jan 26;14(3):406. doi: 10.3390/ani14030406 (PMC10854544; doi:10.3390/ani14030406)
Supplement: Supplementary file 1 [file animals-14-00406-s001.zip › animals-2712303-Supplementary Material.pdf]

Supplementary Material

1. Supplementary Figure S1

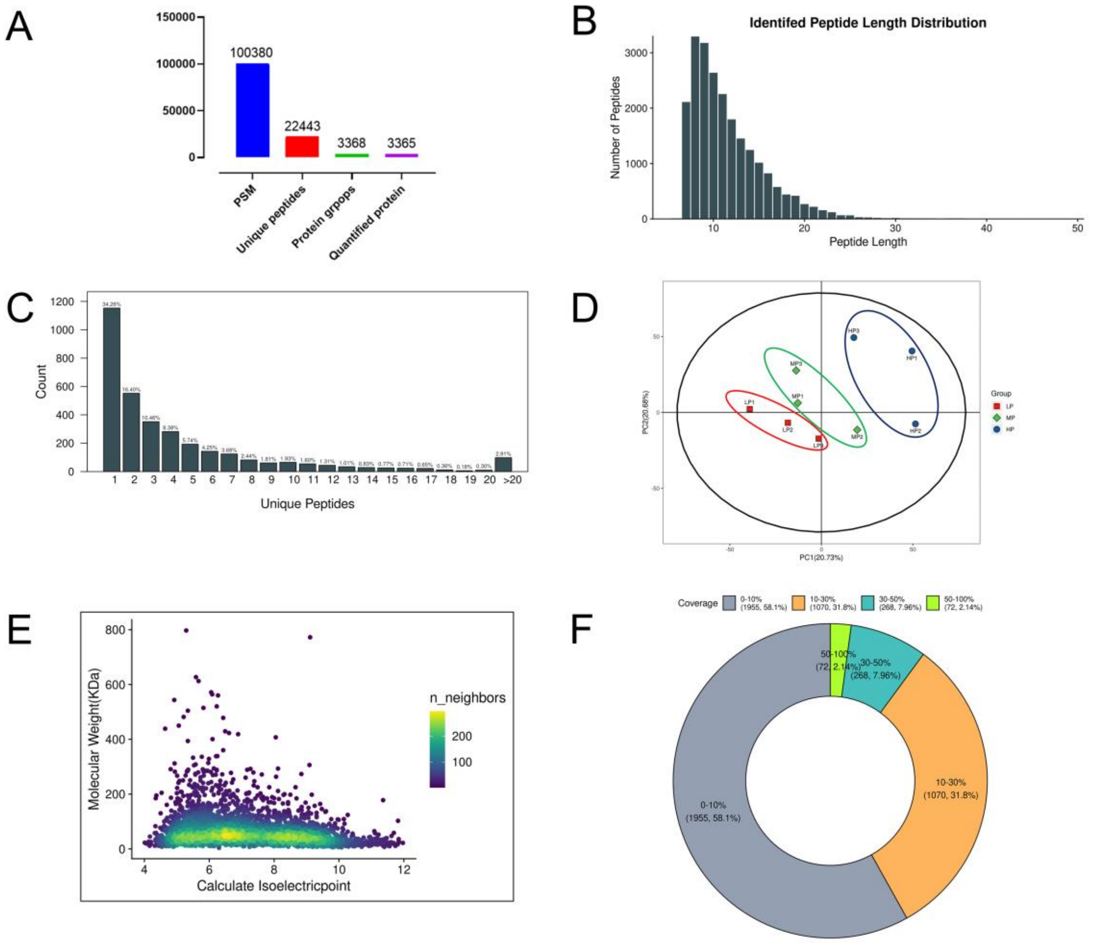

Figure S1: Basic information of proteomic. (A) Spectrums, peptides and identified proteins in LL muscle of Jersey-yak. (B) Identified peptide length distribution. (C) Number of peptides matched to proteins. (D) Principal component analysis. (E) Protein molecular weight and isoelectric point distribution. (F) Protein coverage distribution map.

2. Supplementary Figure S2

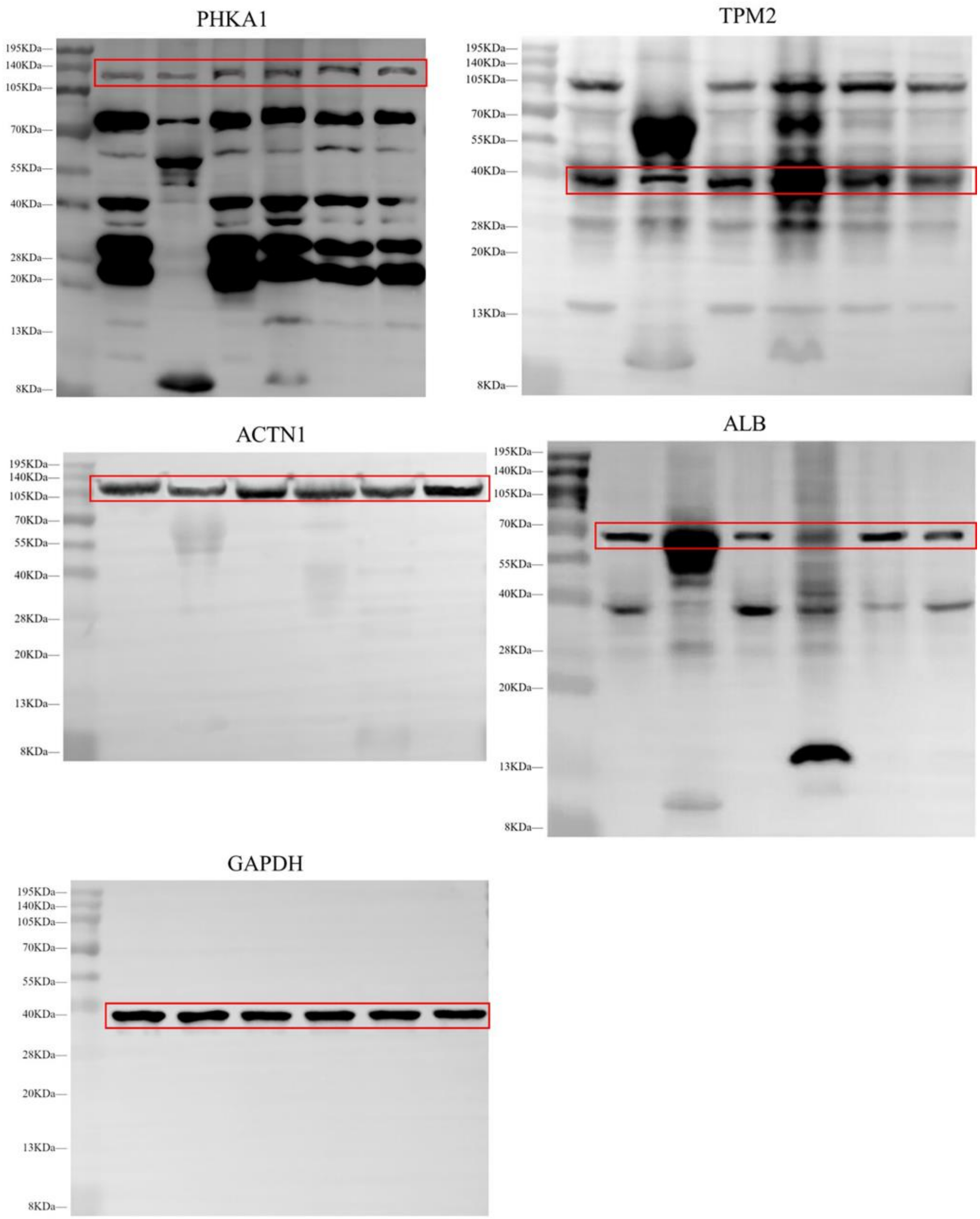

Figure S2: Full-length blots of cropped images presented in manuscript Figure 7. Gel bands cropped are highlighted with red color box.
